# Comparing administrative and survey data for ascertaining cases of irritable bowel syndrome: a population-based investigation

**DOI:** 10.1186/1472-6963-10-31

**Published:** 2010-02-01

**Authors:** Lisa M Lix, Marina S Yogendran, Souradet Y Shaw, Laura E Targownick, Jennifer Jones, Osama Bataineh

**Affiliations:** 1 School of Public Health, University of Saskatchewan, Saskatoon, Canada; 2 Manitoba Centre for Health Policy, University of Manitoba, Winnipeg, Canada; 3 Department of Community Health Sciences, University of Manitoba, Winnipeg, Canada; 4 Section of Gastroenterology, Department of Internal Medicine, University of Manitoba, Winnipeg, Canada; 5 College of Medicine, University of Saskatchewan, Saskatoon, Canada; 6 Department of Mathematics and Statistics, University of Saskatchewan, Saskatoon, Canada

## Abstract

**Background:**

Administrative and survey data are two key data sources for population-based research about chronic disease. The objectives of this methodological paper are to: (1) estimate agreement between the two data sources for irritable bowel syndrome (IBS) and compare the results to those for inflammatory bowel disease (IBD); (2) compare the frequency of IBS-related diagnoses in administrative data for survey respondents with and without self-reported IBS, and (3) estimate IBS prevalence from both sources.

**Methods:**

This retrospective cohort study used linked administrative and health survey data for 5,134 adults from the province of Manitoba, Canada. Diagnoses in hospital and physician administrative data were investigated for respondents with self-reported IBS, IBD, and no bowel disorder. Agreement between survey and administrative data was estimated using the κ statistic. The χ^2 ^statistic tested the association between the frequency of IBS-related diagnoses and self-reported IBS. Crude, sex-specific, and age-specific IBS prevalence estimates were calculated from both sources.

**Results:**

Overall, 3.0% of the cohort had self-reported IBS, 0.8% had self-reported IBD, and 95.3% reported no bowel disorder. Agreement was poor to fair for IBS and substantially higher for IBD. The most frequent IBS-related diagnoses among the cohort were anxiety disorders (34.4%), symptoms of the abdomen and pelvis (26.9%), and diverticulitis of the intestine (10.6%). Crude IBS prevalence estimates from both sources were lower than those reported previously.

**Conclusions:**

Poor agreement between administrative and survey data for IBS may account for differences in the results of health services and outcomes research using these sources. Further research is needed to identify the optimal method(s) to ascertain IBS cases in both data sources.

## Background

Both administrative and survey data are used to conduct population-based health services and outcomes research about chronic conditions such as diabetes, hypertension, arthritis, and osteoporosis [1-5]. A primary concern when using administrative data for studies of chronic conditions is the accuracy and completeness of diagnostic information [6,7], while recall bias is a key concern for self-report data. These limitations of both data sources may result in discrepant research findings, yet only a few studies have compared administrative and survey data for ascertaining disease cases.

Okura et al. [8] observed good agreement between survey and administrative data for ascertaining cases of diabetes, hypertension, myocardial infarction, and stroke. Estimates of agreement were substantially lower for heart failure. Rector et al. [9] found that the sensitivity of administrative data, when compared to survey data, was very good for identifying cases of hypertension and diabetes, but lower for arthritis and heart failure. Other studies have found moderate to good agreement between administrative and survey data [4,10-12]. While agreement tends to be highest for well-defined chronic conditions, such as diabetes, it also depends on the case-ascertainment methodology applied to administrative data [11,12]. For example, Rector et al. [9] found that the number of years of data used to identify chronic disease cases could have a substantial impact on the sensitivity of administrative data.

Recently, administrative data have been investigated as a potential data source for health services and outcomes research about irritable bowel syndrome (IBS) [13]. Population-based investigations about IBS have primarily been conducted using survey data [14-16]. IBS is a common non-inflammatory gastrointestinal condition; the prevalence in North America is estimated to be between 7% and 15% [15-17]. Individuals with IBS have higher health care use and costs than their non-IBS counterparts [18,19]. IBS is a difficult condition to investigate in population-based research because there is no single diagnostic test to confirm disease presence and the symptoms of IBS may also be associated with other conditions, including infections. However, when compared with clinical data or medical charts, diagnoses in administrative data have shown good specificity for ascertaining IBS cases [20,21].

This study compares administrative and survey data for ascertaining cases of IBS. The objectives are to: (1) estimate agreement between the two data sources for IBS and compare the results to those for inflammatory bowel disease (IBD), a gastrointestinal condition with well-defined diagnostic criteria in comparison with IBS; (2) compare the frequency of IBS-related diagnoses in administrative data for survey respondents with and without self-reported IBS, and (3) estimate IBS prevalence from both sources.

## Methods

A retrospective cohort study was undertaken using administrative and survey data from the province of Manitoba, Canada, which has a population of approximately 1.2 million. The Research Data Repository housed at Manitoba Centre for Health Policy (MCHP) contains administrative data provided by the provincial health ministry. The Repository also houses population-based survey data from a national health survey, the Canadian Community Health Survey (CCHS; http://www.statcan.gc.ca/concepts/health-sante/cycle3_1/index-eng.htm) and the two sources can be directly linked via a unique, anonymized personal health identification number (PHIN). The University of Manitoba Health Research Ethics Board approved the conduct of this research and the Manitoba Health Information Privacy Committee approved the data linkage.

The survey data were collected between January and December 2005. The CCHS was developed to provide cross-sectional estimates of health status, health determinants, and health system use for a target population of individuals 12 years of age and older living in private dwellings. The survey does not include individuals living on Indian Reserves and other government-owned land, institutional residents, and full-time members of the Canadian Forces; these groups represent approximately 2% of the Manitoba population. The Manitoba response rate was 83.3%; the national response rate was 78.9%.

Sources of administrative data were records of inpatient hospitalizations and outpatient physician billing claims. Manitoba, like other Canadian provinces, has a universal health system; almost all Manitoba residents are covered under the Manitoba Health Services Insurance Plan (MHSIP). A hospitalization record is completed upon patient discharge. Each record includes up to 16 diagnosis codes from the International Classification of Diseases, 9^th ^Revision, Clinical Modification (i.e., ICD-9-CM) prior to April 1, 2004 and up to 25 diagnosis codes from the 10th Revision for this date onward (i.e., ICD-10-CA). Procedure codes are also captured in hospitalization records. Physicians paid on a fee-for-service basis submit billing claims to the provincial health ministry; these claims capture virtually all outpatient services, including those for hospital emergency departments and outpatient departments. While some physicians are salaried (e.g., approximately 7% of family physicians) [22], it has been estimated that approximately 90% of these physicians submit parallel billing claims for administrative purposes. Each visit results in one billing claim that contains a single ICD-9-CM code. ICD-9 codes in physician data are recorded to the third digit, while codes in hospital data are recorded using up to five digits.

### Study Cohort

There were 7,004 Manitoba respondents to the CCHS in 2005. The anonymized linkage of survey and administrative data was conducted for those respondents who provided their consent for the linkage (*N *= 6,349; 90.6%). After removing invalid or missing PHINs, linkage was successfully achieved for 6,232 respondents (89.0%). From this sample, an adult cohort (19+ years) with at least three years of continuous coverage under the MHSIP prior to the date of their CCHS interview and at least one year of coverage after this date was created (*N *= 5,134; 73.3%). Coverage information was determined from the population registration file.

The survey interview schedule included the following directions: "*Now I'd like to ask about certain chronic health conditions which you may have. We are interested in 'long-term conditions' that have lasted or are expected to last six months or more and that have been diagnosed by a health professional*". Respondents were asked whether they had ever been diagnosed with a number of chronic conditions, including a bowel disorder, specifically: "*Do you suffer from a bowel disorder such as Crohn's disease, ulcerative colitis, irritable bowel syndrome, or bowel incontinence?*" If the response was affirmative, respondents were asked to identify the type of bowel disorder with which they had been diagnosed: Crohn's disease, ulcerative colitis, irritable bowel syndrome, bowel incontinence, or other bowel disorder (type was not specified). A single category was recorded for each respondent.

### Selection of Diagnosis Codes in Administrative Data

Table 1 provides a listing of the diagnosis and procedures codes in administrative data that were selected for this investigation [13,20,21]. Previous studies that have used administrative data to ascertain IBS cases have only been conducted using ICD-9 codes. In this study it was also necessary to identify the relevant codes in ICD-10-CA. This was accomplished using crosswalk files, which map ICD-9 codes to ICD-10-CA codes, developed by a national health information agency, the Canadian Institute of Health Information, and confirmed by research team members.

**Table 1:** Diagnosis and procedure codes selected for the study

Description	ICD-9-CM Diagnosis & Procedure Codes	ICD-10-CA Diagnosis & Procedure Codes
Irritable bowel syndrome	564.1	K58
***Other bowel disorders***		
Crohn's disease	555	K50
Ulcerative colitis	556	K51
***IBS-related diagnoses***		
Gastric ulcer	531	K25
Duodenal ulcer	532	K26
Peptic ulcer	533	K27
Gastrojejunal ulcer	534	K28
Gastritis and duodenitis	535	K29
Disorders of functions of the stomach	536	K30, K31.0, K31.8, K31.9, R11
Other disorders of stomach and duodenum	537	K31.1 - K31.7
Appendicitis	540, 543	K35, K38.0, K38.8
Vascular insufficiency of intestine	557	K55
Intestinal obstruction	560	K56
Diverticula of intestine	562	K57
Necrosis of liver	570	K72
Chronic liver disease	571	K70, K73.9, K73.8, K74.5, K76.0, K76.9
Liver abscess & sequelae	572	K75.0, K75.1, K72.9, K76.6, K76.7, K76.8
Other disorders of liver	573	K76.1, K77.0, K75.9, K76.3, K76.8, K76.9
Cholelithiasis	574	K80
Other disorders of gallbladder	575	K81.0, K81.8, K82
Other disorders of biliary tract	576	K91.5, K83
Diseases of pancreas	577	K85, K86.1, K86.2, K86.8, K86.9
Intestinal malabsorption	579	K90, K91.2
Symptoms: digestive system	787	R11, R12, R13, R14, R15
Symptoms: urinary system	788	N23, N39.4, R30, R32, R33, R34, R35, R36, R39
Symptoms: abdomen and pelvis	789	R10, R16, R18, R19
Endometriosis	617	N80
Pain and other symptoms: female genital organs	625	N94
Anxiety disorders	300	F34.1, F40.1, F40.2, F41.0, F41.1, F41.8, F41.9, F42.8, F44.8, F44.9, F45.2
Intestinal infections: other organisms	008	A04, A08, A02
Ill-defined intestinal infections	009	A09
***IBS-related procedures***		
Sigmoidoscopy, colonoscopy, endoscopy	45.21, 45.22, 45.23, 45.24, 45.28, 48.23	2.NM.70, 2.OW.70
Small bowel series	87.63	3.NK.10

The ICD-9-CM diagnosis code for IBS is 564.1; the corresponding code in ICD-10-CA is K58. Other diagnosis and procedure codes that are more common among individuals with IBS than among individuals without this condition were identified from previous research [13,20,21]. These diagnosis codes were for a variety of gastrointestinal and genitourinary conditions and symptoms, procedures used to assess the presence of gastrointestinal inflammation, and some comorbid conditions. Diagnosis codes for IBD, specifically for Crohn's disease (CD) and ulcerative colitis (UC) were included because some individuals with IBD may have previously been diagnosed with IBS [23]. Diagnosis and procedure codes were investigated in administrative data up to three years prior to the date of the CCHS interview and one year following this date. These time frames were selected based on previous research which has used between one and three years of administrative data to ascertain chronic diseases [12,24].

Crude IBS prevalence estimates, as well as sex- and age-specific estimates, were generated from administrative and survey data. In the administrative data, two case-ascertainment algorithms were investigated. For the first, Manitoba health insurance registrants were identified as IBS cases if they had a least one IBS diagnosis in hospital or physician data in a one-year period. For the second method, registrants were identified as IBS cases if they had at least one IBS diagnosis in a three-year period. One-year estimates were based on data for the 2004/05 fiscal year and three-year estimates were based on data from fiscal years 2002/03 to 2004/05. The population registration file was used to obtain the denominator for these estimates.

### Statistical Analysis

Descriptive statistics, including frequencies and percentages, were used to characterize the cohort on demographic and socioeconomic variables. The *κ *statistic, a chance-corrected measure of agreement, was estimated from the administrative and survey data for both IBS and IBD and 95% confidence intervals (CIs) were computed. The interpretation of *κ *used is [25]: poor agreement (*κ *< 0.20), fair agreement (*κ *= 0.20 to 0.39), moderate agreement (*κ *= 0.40 to 0.59), good agreement (*κ *= 0.60 to 0.79), and very good agreement (*κ *= 0.80 to 1.00). The χ^2 ^test was used to test the association between the frequency of IBS-related diagnoses and procedures in administrative data and self-reported IBS. Ninety-five percent CIs were calculated for the prevalence estimates assuming a binomial distribution. Survey sampling weights were used in all inferential analyses; these weights ensure that the results are generalizable to the Manitoba population. Analyses were performed using SAS software [26].

## Results

Overall, 152 (3.0%) members of the study cohort reported having IBS, another 0.8% reported having CD or UC, and 95.3% reported having no bowel disorder. Another 49 respondents, who indicated they had bowel incontinence or another, unspecified bowel disorder, were excluded from the analysis.

The socio-demographic characteristics of the three groups are described in Table 2. Males comprised less than 15.0% of respondents with self-reported IBS, more than one third of respondents with self-reported CD or UC, and almost half of respondents with no bowel disorder. Almost half of respondents with self-reported IBS were less than 45 years of age compared to slightly more than one-third of those who reported no bowel disorder. The majority of respondents were urban residents. Respondents were less likely to be in the highest and lowest income quintiles and more likely to be in one of the three middle income categories.

**Table 2:** Characteristics of study cohort

Characteristic		Self-Reported IBS (*n *= 152)		Self-Reported CD or UC (*n *= 41)		No Reported Bowel Disorder (*n *= 4,892)	
		***n***	***%***	***n***	***%***	***n***	***%***
		
Sex	Male	22	14.4	15	36.5	2,258	46.2
	Female	130	85.6	26	63.5	2,634	53.8
Age Group	< 45 years	68	44.7	18	43.9	1,916	39.2
	45 - 64 years	63	41.5	14	34.1	1,693	34.6
	65+ years	21	13.8	9	22.0	1,283	26.4
Region of Residence	Urban	105	69.1	29	70.7	3,156	64.5
	Rural	47	30.9	12	29.3	1,736	35.5
Income Quintile^a^	Q1 (lowest)	20	13.2	--	--	809	16.5
	Q2	35	23.0	--	--	1014	20.7
	Q3	36	23.7	13	31.7	1126	23.0
	Q4	36	23.7	8	19.5	865	17.7
	Q5 (highest)	23	15.1	9	22.0	948	19.4

Note: IBS - Irritable bowel syndrome; CD - Crohn's disease; UC - Ulcerative colitis; -- indicates that the results have been suppressed due to cell sizes less than 7.

^a^Percentages for this variable may not sum to 100 because of missing data

Estimates of agreement between survey and administrative data are reported in Table 3. Applying the interpretative criteria to these estimates [25], agreement was poor for IBS when one year of post-interview data was used for case ascertainment but was fair when three years of pre-interview data were used. For IBD, agreement was moderate when a single year of post-interview data was used, but was good when up to three years of administrative data prior to the interview date were used.

**Table 3:** Estimates of *κ *for administrative and survey data before and after the survey interview date

Condition	3 Years Before	1 Year After
Self-Reported IBS	0.22 (0.22, 0.23)	0.11 (0.11, 0.12)
Self-Reported CD or UC	0.65 (0.64, 0.66)	0.48 (0.46, 0.49)

Note: IBS - Irritable bowel syndrome; CD - Crohn's disease; UC - Ulcerative colitis; 95% confidence intervals are provided in parentheses.

Table 4 reports the percentage of survey respondents with self-reported IBS and no self-reported bowel disorder for the selected diagnoses and procedures in administrative data before and after the survey interview date. None of the survey respondents had an IBS or IBS-related diagnosis code recorded in hospitalization records. An IBD diagnosis in hospitalization records was also rare among self-reported IBS cases. There was a significant association between the presence of a sigmoidoscopy, colonoscopy, or endoscopy procedure in both three-year and one-year periods before the survey interview and presence of self-reported IBS (*p *< 0.001). Overall, 11.2% of survey respondents who reported a diagnosis of IBS had one of the selected procedure codes in the three years prior to the interview date. In physician billing claims, 9.4% of the self-reported IBS cases had an IBS diagnosis prior to the interview date and a similar percentage (10.7%) had a diagnosis in the one-year period following this date. An IBS diagnosis code was present in the physician claims of less than 2.0% of respondents who reported that they did not have a bowel disorder.

**Table 4:** Percentages of respondents with selected diagnoses and procedures before and after the survey interview date

Diagnosis/Procedure	Self-Reported IBS (*n *= 152)			No Reported Bowel Disorder (*n *= 4,892)		
	
	3 Years Before	1 Year Before	1 Year After	3 Years Before	1 Year Before	1 Year After
	%	%	%	%	%	%

***Hospitalization records***						
IBS	0.0	0.0	0.0	0.0	0.0	0.0
CD or UC	--	0.0	0.0	0.2	0.0	0.0
All IBS-related diagnoses	0.0	0.0	0.0	0.0	0.0	0.0
***Procedures***						
Sigmoidoscopy, colonoscopy, endoscopy	11.2*	3.8*	1.6^†^	4.1	1.5	1.7
Small bowel series	0.0	0.0	0.0	0.0	0.0	0.0
***Physician claims***						
IBS	9.4*	9.4*	10.7*	1.0	1.0	1.5
CD/UC	22.5*	--	--	4.7	1.8	1.7
Gastric ulcer	--	--	0.0	--	--	0.0
Duodenal ulcer	--	--	0.0	--	--	0.0
Peptic ulcer	1.2^†^	--	--	0.9	0.3	0.3
Gastrojejunal ulcer	0.0	0.0	0.0	0.0	0.0	0.0
Gastritis and duodenitis	7.5*	3.5*	2.1*	3.4	1.2	1.1
Disorders of functions of the stomach	3.3*	--	1.9*	2.2	0.8	0.8
Other disorders of stomach and duodenum	--	--	0.0	0.0	--	0.0
Appendicitis	--	--	0.0	0.0	0.1	0.2
Vascular insufficiency of intestine	--	0.0	0.0	0.0	0.0	0.0
Intestinal obstruction	2.7*	--	0.0	0.5	0.2	0.1
Diverticula of intestine	10.6*	--	--	1.0	0.2	0.3
Necrosis of liver	0.0	0.0	0.0	0.0	--	0.0
Chronic liver disease	--	--	--	0.2	0.1	0.1
Liver abscess & sequelae	0.0	0.0	0.0	0.0	0.0	0.0
Other disorders of liver	--	--	0.0	0.4	0.1	0.5
Cholelithiasis	0.0	0.0	0.0	0.0	0.0	0.0
Other disorders of gallbladder	0.0	0.0	0.0	0.4	0.1	0.2
Other disorders of biliary tract	0.0	0.0	0.0	0.2	--	0.0
Diseases of pancreas	0.0	0.0	0.0	0.0	0.1	0.0
Intestinal malabsorption	0.0	0.0	0.0	0.3	--	0.1
Symptoms: digestive system	8.4*	2.1	--	3.2	1.1	1.6
Symptoms: urinary system	4.3*	2.9*	4.2*	2.8	1.0	1.4
Symptoms: abdomen & pelvis	26.9*	12.9*	10.2*	12.9	4.9	5.6
Endometriosis	--	--	--	0.4	--	0.3
Pain and other symptoms, female genital organs	5.6*	--	--	3.1	1.5	1.9
Anxiety disorders	34.4*	19.7*	20.7*	19.3	9.4	8.3
Intestinal infections due to other organism	--	--	--	0.6	0.3	0.3
Ill-defined intestinal infections	--	--	0.0	0.5	0.1	0.3

Note: IBS = irritable bowel syndrome; CD = Crohn's disease; UC = ulcerative colitis; -- indicates results have been suppressed due to cell sizes less than 7; * indicates a χ^2 ^test for IBS respondents and respondents with no bowel disorder that is significant at *a *= .05) and † indicates a χ^2 ^test that is non-significant. Unmarked numeric values for IBS respondents could not be tested due to zero/small cell sizes.

Almost one-quarter of IBS cases had a diagnosis of IBD in physician claims data up to three years before the survey interview date, but in the year immediately before or after the interview date the number of cases with this diagnosis was too small to analyze. Among IBS cases, the most frequent IBS-related diagnoses recorded in physician billing claims in the three-year period before the survey interview date were anxiety disorders (34.4%), symptoms involving the abdomen and pelvis (26.9%), and diverticulitis of the intestine (10.6%). In the one-year period following the interview date the most common IBS-related diagnoses were anxiety disorders (20.7%), symptoms involving the abdomen and pelvis (10.2%), and symptoms involving the urinary system (4.2%). These were also the most common IBS-related diagnoses among non-cases, but the percentages were significantly different (*p *< 0.001 for all tests) for the two groups.

The crude IBS prevalence estimate from survey data was 3.00 per 100 (95% CI: 2.37, 3.76). Using administrative data, the crude prevalence estimate was 1.71 per 100 (95% CI: 1.69, 1.74) for a one-year case-ascertainment algorithm and 4.23 per 100 (95% CI: 4.19, 4.28) for a three-year algorithm. The number of IBS cases identified from hospitalization data was small; for the former algorithm, 1.74% of cases were identified solely from hospital data and for the latter algorithm the corresponding figure was 0.43% of cases.

**Figure 1:**
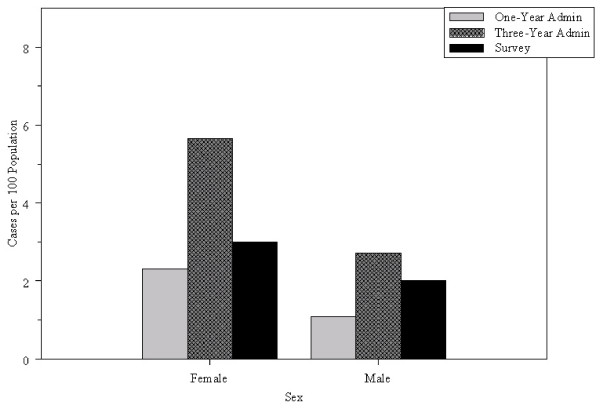
**Sex-specific estimates of IBS prevalence from survey and administrative data**.

**Figure 2:**
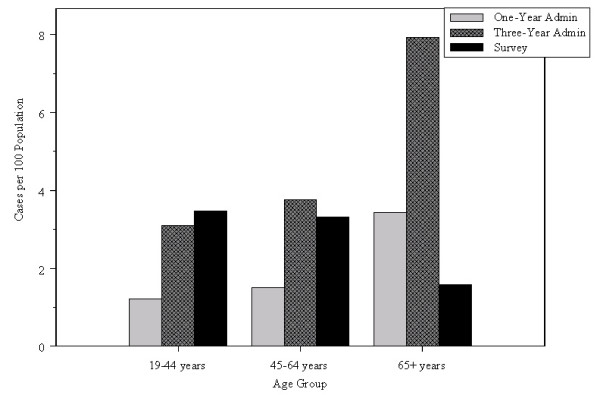
**Age-specific estimates of IBS prevalence from survey and administrative data**.

Both administrative and survey data resulted in higher prevalence estimates for females than for males (Figure 1); the ratio was 4.27 for survey data, 2.14 for the one-year algorithm and 2.08 for the three-year algorithm. The analysis by age group (Figure 2) revealed that for the two youngest age groups, prevalence estimates from administrative and survey data were not significantly different (p ≤ .10 for both administrative data algorithms). For the oldest age group, they were significantly higher using administrative data than survey data.

## Discussion

This study compared population-based administrative and survey data for IBS diagnoses. The results show that agreement between administrative and survey data for IBS was low, but it was much higher for IBD. Agreement was investigated for two periods of time both before and after the survey interview date. Agreement between administrative and survey data remained low regardless of the size or direction (i.e., pre-interview versus post-interview) of the case-ascertainment window.

Compared with survey respondents who reported having no bowel disorder, respondents with a self-reported diagnosis of IBS were more likely to have diagnoses in administrative data for symptoms of the digestive system and abdomen, other gastrointestinal conditions including IBD, selected procedures, and an anxiety disorder. These findings are consistent with previous research, which has shown that that IBS patients have an increased likelihood of diagnosis for a variety of gastrointestinal, genitourinary, and psychological conditions when compared with the general population [21,27]. Given that IBD and IBS may have similar symptoms, an increased frequency of diagnosis for IBD may be indicative of physicians attempting to "rule out" the presence of this condition in IBS patients [23]. A recent Canadian study [28] found that physicians are accurate in their use of ICD-9 diagnosis codes for Crohn's disease and ulcerative colitis in billing claims. Thus, the increased frequency of IBD diagnosis among IBS patients does not appear to be due to inaccuracies in diagnoses.

IBS prevalence estimates have been reported to be two to three times higher among females than males [17], which is consistent with the findings observed in this study for both data sources. However, crude estimates in this study were still lower than those reported in previous North American research [14,16,17]. This may be due to a number of factors, including the lack of specificity of the survey questions about bowel disorders or the unfamiliarity of Manitoba physicians with diagnostic criteria for IBS. However, there is substantial international variation in IBS prevalence estimates, with some non-North American counties reporting values as low as 3 and 5 per 100 [29]. The estimates obtained from administrative data were higher for the oldest age group than for the younger two age groups, which is not consistent with the estimates obtained from survey data nor with the estimates reported in previous research, which show declining IBS prevalence with age [15]. This may be a result of some loss of specificity for ascertaining IBS cases in physician data because diagnoses in Manitoba's physician data are only recorded to the third digit; very few IBS cases were identified from hospital data, in which diagnoses are recorded with greater specificity. This finding may also be a result of increased reporting among older adults of a variety of symptoms and/or an increased likelihood of inaccurate assignment of an IBS diagnosis in older adults [30].

Well-defined chronic conditions are more likely to result in good agreement between administrative and survey data [8,31]. The Rome criteria have been developed to provide physicians with a systematic methodology to classify individuals with functional gastrointestinal disorders, including IBS [32]. However, based on the results of medical chart review, Goff et al. [20] reported that the criteria are infrequently used by physicians to establish a diagnosis of IBS. Wilson et al.[16] suggest that the use of the Rome criteria by physicians may result in underestimation of IBS prevalence in population-based research that uses medical records.

One limitation of this study is that it was not possible to compare clinical data with the administrative data or survey data. As well, the survey data do not contain information about date of diagnosis, which would have been useful for decisions about the number of years of administrative data needed to ascertain disease cases. Some diagnoses occurred infrequently in administrative data and therefore it was not possible to test for differences between survey respondents with and without IBS. Finally, as noted previously, only three-digit ICD-9 codes are available in physician claims data in Manitoba, resulting in loss of specificity to ascertain IBS cases. This situation is not unique in Canada; physician data in Ontario, the largest province in Canada, is also limited to three-digit diagnosis codes [33]. However, there is a lack of consistency across jurisdictions in the way diagnoses are recorded in physician data. For example, ICD-9 codes in Medicare physician data from the United States may be recorded using up to five digits [9].

Given the low agreement between survey and administrative data and evidence that this disease appears to be under-reported in both data sources, it is important to undertake further research that can improve IBS case ascertainment in population-based data. Thompson et al. [32] suggest that imprecision in the wording of survey questions contributes to inaccuracies in case ascertainment. Wilson et al. [16] recommended that survey questions based on the Rome criteria be supplemented with additional questions to identify patients formerly diagnosed with IBS but with symptoms currently under control. Thus, future research could focus on comparing agreement between administrative and survey data when different question wording methods are adopted. For administrative data, techniques that do not rely exclusively on a single diagnosis code could be used to improve ascertainment results for IBS. Machine-learning and statistical classification models, including latent class analysis and neural networks, which have been applied to chronic conditions where there may be low sensitivity associated with using a single diagnosis code to ascertain disease cases [12,34,35], might be applied to this problem.

## Conclusions

Population-based chronic disease research can provide important information about the effectiveness of disease treatment and management initiatives and health promotion and disease prevention strategies. The quality of this research depends, in part, on the accuracy and validity of chronic disease case ascertainment methods. In both data sources, the use of a single methodology for identifying disease cases may result in missed cases. Finally, researchers who rely on either administrative data or survey data to conduct population-based studies about IBS should recognize that lack of comparability between the two data sources will be an important confounder of their results.

## Competing interests

The authors declare that they have no competing interests.

## Authors' contributions

LML, MSY, SYS, LET, JJ, and OB contributed equally to this paper. LML, MSY, SYS, and LET planned the study. MSY carried out the analysis and OB summarized the data. LML, MSY, SYS, LET, JJ, and OB drafted the manuscript. All authors have read and approved the final manuscript.

## Pre-publication history

The pre-publication history for this paper can be accessed here:

http://www.biomedcentral.com/1472-6963/10/31/prepub
